# Comparative Effects of Mineral Oil, Corn Oil, Eicosapentaenoic Acid, and Docosahexaenoic Acid in an In Vitro Atherosclerosis Model

**DOI:** 10.1161/JAHA.122.029109

**Published:** 2023-03-21

**Authors:** Samuel C. R. Sherratt, Peter Libby, Deepak L. Bhatt, R. Preston Mason

**Affiliations:** ^1^ Department of Molecular, Cellular, and Biomedical Sciences University of New Hampshire Durham NH USA; ^2^ Elucida Research LLC Beverly MA USA; ^3^ Department of Medicine, Cardiovascular Division, Brigham and Women’s Hospital Harvard Medical School Boston MA USA; ^4^ Mount Sinai Heart Icahn School of Medicine at Mount Sinai New York NY USA

**Keywords:** corn oil, docosahexaenoic acid, eicosapentaenoic acid, lipid oxidation, mineral oil, Basic Science Research, Lipids and Cholesterol, Atherosclerosis

Therapeutic administration of omega‐3 fatty acids has yielded discordant results in clinical trials.^1^ In the Reduction of Cardiovascular Events with Icosapent Ethyl‐Intervention Trial (REDUCE‐IT), treatment with icosapent ethyl (IPE), an ethyl ester of the active ingredient eicosapentaenoic acid (EPA, 20:5; n−3), at 4 g/d to patients with high cardiovascular risk showed a 25% relative risk reduction of the primary end point (5‐point composite major adverse cardiac event) compared with placebo.^2^ By contrast, the Long‐Term Outcomes Study to Assess Statin Residual Risk with Epanova in High Cardiovascular Risk Patients with Hypertriglyceridemia (STRENGTH) trial, which used a mixed combination of EPA/docosahexaenoic acid (DHA, 22:6; n−3) free carboxylic acids (4 g/d) in a similar high cardiovascular risk population, did not reduce the same primary end point compared with placebo.

Despite their distinct formulations, some have attributed these large differences in clinical outcomes to placebo choice: pharmaceutical grade mineral oil (MO; REDUCE‐IT) or corn oil (CO; STRENGTH). It has been suggested that MO even at 2 mL BID interferes with statin absorption or promotes intestinal inflammation compared with CO, although laboratory studies have failed to substantiate these claims.^1^

A recent substudy of REDUCE‐IT showed small but statistically significant absolute increases in several inflammatory and lipid biomarkers, including high‐sensitivity C‐reactive protein, in the placebo arm compared with baseline, while changes in the IPE arm were minimal.^3^ These placebo oils differ in composition: MO contains saturated, aliphatic hydrocarbons ranging from ≈C15 to C26, while CO is primarily composed of linoleic acid (18:2; n−6). To explore any potential impact of placebo choice on clinical outcomes, we asked whether MO affects certain atherogenic mechanisms differently than CO, EPA, or DHA.

We therefore compared the effects of pharmaceutical grade MO, CO, EPA, and DHA on rates of lipoprotein and membrane oxidation. Lipoproteins transport dietary long‐chain fatty acids that may influence rates of oxidation, a process implicated in atherosclerosis. Membrane oxidation leads to loss of protein function and normal signal‐transduction. We used pharmacologic concentrations of EPA and compared with equimolar levels of DHA, MO, and CO (10 μmol/L) or vehicle. Both the MO and CO met United States Pharmacopeia testing specifications.

Small‐dense low‐density lipoprotein (sdLDL) and very low‐density lipoprotein were isolated from human plasma by isopycnic centrifugation. Each subfraction was separated into samples of 50 to 100 μg/mL, respectively, and incubated at 37 °C for 30 minutes with each treatment or equivolume vehicle. Samples were then subjected to copper‐induced oxidation (20 μmol/L) with oxidation monitored by malondialdehyde formation.^4^ Model membranes were prepared as binary mixtures of 1,2‐dilinoleoyl‐*sn*‐glycero‐3‐phosphocholine and cholesterol at a 0.6 cholesterol‐to‐phospholipid mole ratio with each treatment and exposed to oxidative conditions as previously described.^4^ Statistical analyses were performed using GraphPad InStat v3.10 for Windows. For multiple comparisons, statistical significance was determined using ANOVA followed by Tukey–Kramer multiple comparisons post hoc analysis. Alpha error was set to 0.05. Institutional review board approval was not required for this study. The data supporting the findings of this study will be made available by the corresponding author upon reasonable request.

After 4 hours, oxidation increased 15‐fold and 57‐fold (Figure) in vehicle‐treated sdLDL and very low‐density lipoprotein, respectively (*P*<0.001). The oxidation rate was unaffected by MO or CO. By contrast, EPA significantly inhibited sdLDL and very low‐density lipoprotein oxidation by 75% and 94%, respectively, compared with vehicle (*P*<0.001). While DHA exhibited antioxidant activity at 2 hours at a level less than EPA in sdLDL (*P*<0.001), this effect diminished by 4 hours. Similar trends were observed in model membranes. There was no change in the oxidation rate in membranes incubated with either placebo oil through 72 hours. EPA inhibited membrane oxidation by 89% compared with vehicle after 72 hours (*P*<0.001), while DHA inhibited oxidation less effectively (*P*<0.05).

**Figure:**
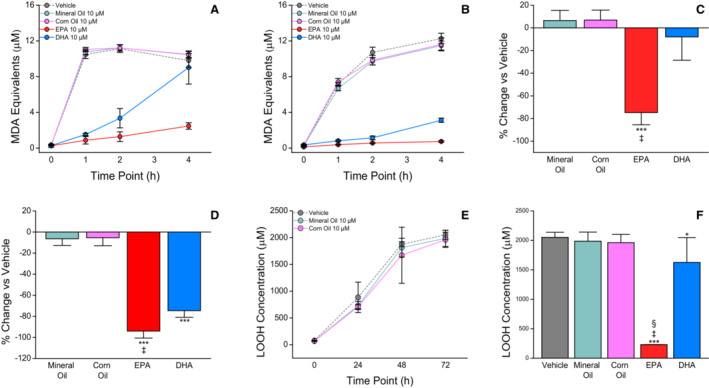
Comparative effects of mineral oil, corn oil, EPA, and DHA on lipoprotein and membrane oxidation. Lipoprotein fractions were oxidized using copper sulfate following incubation with mineral oil, corn oil, EPA, or DHA. Model lipid membranes were exposed to atmospheric conditions to induce oxidation. The sdLDL (**A**) and VLDL (**B**) samples were incubated with mineral oil, corn oil, EPA, or DHA at 10 μmol/L, and oxidation was measured using colorimetric techniques capturing MDA levels, a secondary product of lipid oxidation. EPA significantly reduced oxidation more than DHA through 4 hours in sdLDL (**A**, **C**) and VLDL (**B**, **D**). In model membranes (**E**), oxidation was measured using colorimetric techniques capturing levels of LOOHs, which are a primary product of lipid oxidation. No significant change in the rate of oxidation with 10 μmol/L mineral oil or corn oil, and once again EPA significantly reduced membrane oxidation more than DHA through 72 hours (**F**). **C,** ***P<0.001 vs mineral oil and corn oil; ^‡^ *P*<0.001 vs DHA; (Tukey–Kramer Multiple Comparisons Test; overall ANOVA: *P*<0.0001). Values are mean±SD (N=3–6). **D**, ***P<0.001 vs mineral oil and corn oil; ^‡^ *P*<0.05 vs DHA (Tukey–Kramer Multiple Comparisons Test; overall ANOVA: *P*<0.0001). Values are mean±SD (N=3). **F**, ***P<0.001 vs vehicle; *P<0.05 vs vehicle; ^‡^ *P*<0.001 vs mineral oil, corn oil; ^§^ *P*<0.001 vs DHA (Tukey–Kramer Multiple Comparisons Test, overall ANOVA: *P*<0.0001). Values are mean±SD (N=3–6). DHA indicates docosahexaenoic acid; EPA, eicosapentaenoic acid; LOOH, lipid hydroperoxide; MDA, malondialdehyde; sdLDL, small‐dense low‐density lipoprotein; and VLDL, very low‐density lipoprotein.

Achieved blood levels of EPA were associated with cardiovascular benefit in REDUCE‐IT, compatible with a direct mechanistic effect on atherosclerosis.^1^ Plaque volume measurement techniques found that EPA, but not EPA with DHA, significantly reduced total plaque and lipid volume when added to statins in patients with stable coronary artery disease or acute coronary syndrome.^1^ Laboratory findings have also observed differences between EPA and DHA with respect to cholesterol efflux, endothelial function, and membrane stability.^1^, ^4^ Importantly, EPA and DHA also exhibit antagonistic effects on membrane structure where they concentrate as components of phospholipids.^1^ Such broad physico‐chemical and biological differences may contribute to divergent clinical outcomes between IPE and other O3FA formulations, which is an ongoing area of research.

Pharmaceutical grade MO has often served as a placebo in clinical trials because of its safety profile and defined composition without reproducible patterns of lipid or inflammatory biomarker changes.^5^ Post hoc analyses of REDUCE‐IT by the US Food and Drug Association found that changes in low‐density lipoprotein or high‐sensitivity C‐reactive protein concentrations in the placebo arm did not influence the favorable cardiovascular benefits of IPE.^5^ Combined with the current in vitro data, it seems unlikely that administration of MO promotes atherosclerosis to a degree sufficient to account for the full benefit observed with EPA treatment in REDUCE‐IT.

In conclusion, the current data support potent antioxidant effects for EPA in ApoB‐containing lipoproteins, especially highly atherogenic sdLDL, and membranes that were sustained over time compared with DHA and placebo oils. Along with other distinct mechanisms, EPA antioxidant properties may contribute to reduced cardiovascular events in outcome trials using IPE compared with n3‐FA combinations, independent of placebo selection.

## Sources of Funding

None.

## Disclosures

Mr Samuel C.R. Sherratt has an employment relationship with Elucida Research, which has received research funding from Amarin and the Cleveland Clinic. Dr Peter Libby is an unpaid consultant to, or involved in clinical trials for Amgen, AstraZeneca, Baim Institute, Beren Therapeutics, Esperion Therapeutics, Genentech, Kancera, Kowa Pharmaceuticals, Medimmune, Merck, Novo Nordisk, Novartis, Pfizer, and Sanofi‐Regeneron. Dr Libby is an unpaid consultant to, or involved in clinical trials for Amgen, AstraZeneca, Baim Institute, Beren Therapeutics, Esperion Therapeutics, Genentech, Kancera, Kowa Pharmaceuticals, Medimmune, Merck, Moderna, Novo Nordisk, Novartis, Pfizer, and Sanofi‐Regeneron. Dr Libby is a member of the scientific advisory board for Amgen, Caristo Diagnostics, Cartesian Therapeutics, CSL Behring, DalCor Pharmaceuticals, Dewpoint Therapeutics, Eulicid Bioimaging, Kancera, Kowa Pharmaceuticals, Olatec Therapeutics, Medimmune, Moderna, Novartis, PlaqueTec, TenSixteen Bio, Soley Therapeutics, and XBiotech, Inc. Dr Libby's laboratory has received research funding in the last 2 years from Novartis. Dr Libby is on the Board of Directors of XBiotech, Inc. Dr Libby has a financial interest in Xbiotech, a company developing therapeutic human antibodies, in TenSixteen Bio, a company targeting somatic mosaicism and clonal hematopoiesis of indeterminate potential (CHIP) to discover and develop novel therapeutics to treat age‐related diseases, and in Soley Therapeutics, a biotechnology company that is combining artificial intelligence with molecular and cellular response detection for discovering and developing new drugs, currently focusing on cancer therapeutics. Dr Libby's interests were reviewed and are managed by Brigham and Women's Hospital and Mass General Brigham in accordance with their conflict‐of‐interest policies. Dr Libby receives funding support from the National Heart, Lung, and Blood Institute (1R01HL134892 and 1R01HL163099‐01), the American Heart Association (18CSA34080399), the RRM Charitable Fund, and the Simard Fund. Dr Deepak L. Bhatt discloses the following relationships—Advisory Board: AngioWave, Bayer, Boehringer Ingelheim, Cardax, CellProthera, Cereno Scientific, Elsevier Practice Update Cardiology, High Enroll, Janssen, Level Ex, McKinsey, Medscape Cardiology, Merck, MyoKardia, NirvaMed, Novo Nordisk, PhaseBio, PLx Pharma, Regado Biosciences, Stasys; Board of Directors: AngioWave (stock options), Boston VA Research Institute, Bristol Myers Squibb (stock), DRS.LINQ (stock options), High Enroll (stock), Society of Cardiovascular Patient Care, TobeSoft; Chair: Inaugural Chair, American Heart Association Quality Oversight Committee; Consultant: Broadview Ventures; Data Monitoring Committees: Acesion Pharma, Assistance Publique‐Hôpitaux de Paris, Baim Institute for Clinical Research (formerly Harvard Clinical Research Institute, for the PORTICO trial, funded by St. Jude Medical, now Abbott), Boston Scientific (Chair, PEITHO trial), Cleveland Clinic (including for the ExCEED trial, funded by Edwards), Contego Medical (Chair, PERFORMANCE 2), Duke Clinical Research Institute, Mayo Clinic, Mount Sinai School of Medicine (for the ENVISAGE trial, funded by Daiichi Sankyo; for the ABILITY‐DM trial, funded by Concept Medical), Novartis, Population Health Research Institute; Rutgers University (for the NIH‐funded MINT Trial); Honoraria: American College of Cardiology (Senior Associate Editor, Clinical Trials and News, ACC.org; Chair, ACC Accreditation Oversight Committee), Arnold and Porter law firm (work related to Sanofi/Bristol‐Myers Squibb clopidogrel litigation), Baim Institute for Clinical Research (formerly Harvard Clinical Research Institute; RE‐DUAL PCI clinical trial steering committee funded by Boehringer Ingelheim; AEGIS‐II executive committee funded by CSL Behring), Belvoir Publications (Editor in Chief, Harvard Heart Letter), Canadian Medical and Surgical Knowledge Translation Research Group (clinical trial steering committees), Cowen and Company, Duke Clinical Research Institute (clinical trial steering committees, including for the PRONOUNCE trial, funded by Ferring Pharmaceuticals), HMP Global (Editor in Chief, *Journal of Invasive Cardiology*), *Journal of the American College of Cardiology* (Guest Editor; Associate Editor), K2P (Co‐Chair, interdisciplinary curriculum), Level Ex, Medtelligence/ReachMD (CME steering committees), MJH Life Sciences, Oakstone CME (Course Director, Comprehensive Review of Interventional Cardiology), Piper Sandler, Population Health Research Institute (for the COMPASS operations committee, publications committee, steering committee, and USA national co‐leader, funded by Bayer), Slack Publications (Chief Medical Editor, *Cardiology Today's Intervention*), Society of Cardiovascular Patient Care (Secretary/Treasurer), WebMD (CME steering committees), Wiley (steering committee); Other: *Clinical Cardiology* (Deputy Editor), NCDR‐ACTION Registry Steering Committee (Chair), VA CART Research and Publications Committee (Chair); Patent: Sotagliflozin (named on a patent for sotagliflozin assigned to Brigham and Women's Hospital who assigned to Lexicon; neither I nor Brigham and Women's Hospital receive any income from this patent); Research Funding: Abbott, Acesion Pharma, Afimmune, Aker Biomarine, Amarin, Amgen, AstraZeneca, Bayer, Beren, Boehringer Ingelheim, Boston Scientific, Bristol‐Myers Squibb, Cardax, CellProthera, Cereno Scientific, Chiesi, CinCor, CSL Behring, Eisai, Ethicon, Faraday Pharmaceuticals, Ferring Pharmaceuticals, Forest Laboratories, Fractyl, Garmin, HLS Therapeutics, Idorsia, Ironwood, Ischemix, Janssen, Javelin, Lexicon, Lilly, Medtronic, Merck, Moderna, MyoKardia, NirvaMed, Novartis, Novo Nordisk, Owkin, Pfizer, PhaseBio, PLx Pharma, Recardio, Regeneron, Reid Hoffman Foundation, Roche, Sanofi, Stasys, Synaptic, The Medicines Company, Youngene, 89Bio; Royalties: Elsevier (Editor, Braunwald's Heart Disease); Site Co‐Investigator: Abbott, Biotronik, Boston Scientific, CSI, Endotronix, St. Jude Medical (now Abbott), Philips, SpectraWAVE, Svelte, Vascular Solutions; Trustee: American College of Cardiology; Unfunded Research: FlowCo, Takeda. Dr R. Preston Mason has received grant/research support from Amarin, HLS Therapeutics, Lexicon, and the Cleveland Clinic.
